# Bactériémie à Kingella denitrificans chez un enfant suivi pour un syndrome d’insuffisance médullaire

**DOI:** 10.11604/pamj.2017.28.83.13698

**Published:** 2017-09-27

**Authors:** Abdeslam Hiddou, Yassine Zemmrani, Yassine Ahroui, Nabila Soraa

**Affiliations:** 1 Service de Microbiologie, CHU Mohammed VI, Faculté de Médecine et de Pharmacie, Université Cadi Ayyad, Marrakech, Maroc

**Keywords:** Kingella denitrificans, bactériémie, enfant, Kingella denitrificans, bacteremia, child

## Abstract

*Kingella denitrificans* est un micro-organisme faisant partie de la flore oropharangienne non pathogène. Ce germe est récemment reconnu responsable d'infections invasives opportunistes qui surviennent essentiellement sur des terrains d'immunodépression. Ainsi, Il nous a paru intéressant de rapporter un cas de bactériémie due à *Kingella denitrificans*, bacilles gram négatif de culture difficile encore exceptionnellement décrit dans la littérature chez un enfant âgé de 3 ans et 7 mois suivi depuis l'âge de un an pour un syndrome d'insuffisance médullaire avec pancytopénie dont l'origine est restée indéterminée. Les cliniciens et les microbiologistes doivent penser davantage à ce germe surtout sur un terrain débilité. L'utilisation des flacons d'hémoculture contribue d'une façon significative à la détection de ce germe.

## Abstract (en)

Kingella denitrificans is a non-pathogenic micro-organism present in oropharyngeal flora. This germ has been recently recognized as responsible for opportunistic invasive infections mainly affecting immunosuppressed patients. We here report the case of a child aged 3 years and 7 months followed-up since the age of one year for bone marrow failure syndrome associated with pancytopenia of undetermined origin who had bacteremia due to Kingella denitrificans, a group of difficult to culture gram-negative bacteria rarely described in the literature. Clinicians and microbiologists should suspect the presence of this germ especially in immunosuppressed patients. The use of blood culture bottle contributes in a significant way to the detection of this germ.

## Introduction

Le genre Kingella comprend essentiellement deux espèces rarement isolées en bactériologie clinique: *Kingella kingae* et *Kingella denitrificans*. Ce genre appartient à la famille des Neisseriaceae, et est étroitement lié aux genres Neisseria et Eikenella. Les infections causées par les espèces Kingella sont peu fréquentes, et généralement sont considérées comme des microorganismes commensaux des voies respiratoires supérieures avec un potentiel pathogène faible. *Kingella denitrificans*, coccobacille exigeant à Gram négatif a été récemment reconnu comme responsable d'infections opportunistes invasives, types endocardites, bactériémies et rarement des méningites [1]. Nous décrivons ici l'observation d'un enfant de 3 ans et 7 mois pris en charge pour un syndrome d'insuffisance médullaire d'origine indéterminée avec une pancytopénie, qui s'est compliqué d'une bactériémie à *Kingella denitrificans*.

## Patient et observation

Il s'agissait d'un enfant âgé de 3 ans et 7 mois, issu d'un mariage consanguin 1^er^ degré, qui est suivi depuis l'âge d'un an pour un syndrome d'insuffisance médullaire d'origine indéterminée avec une pancytopénie dont le bilan étiologique était non concluant. Il a été transfusé à plusieurs reprises à raison d'une fois par mois. L'enfant ayant comme antécédents une sœur décédée à l'âge de 18 mois pour maladie de Byler. Le début de la symptomatologie remonte à 3 jours de la date de la consultation, par l'installation brutale d'une fièvre associée à des diarrhées glaireuses non sanglantes à raison de 8 selles par jours sans vomissements ni douleurs abdominales ou autres signes infectieux notamment pulmonaires. Le tout évoluant dans un contexte de fièvre chiffrée à 39-40°, et d'une intense pâleur cutanéomuqueuse avec altération de l'état générale. L'examen clinique a trouvé un enfant conscient tachycarde à 120bpm et polypnéique à 40cpm avec un syndrome infectieux (fièvre 39-40°), un syndrome anémique (intense pâleur cutanéomuqueuse, asthénie) sans syndrome hémorragique et sans autre anomalies notamment pas de syndrome tumoral ou adénopathie et avec un tableau de déshydratation sévère. A côté du bilan paraclinique standard fait de NFS (Hb: 6.2 g/dl, VGM:76 fL, TCMH:27 pg, GB: 2300, PNN 390 /mm^3^, Lymphocytes à 1850 Plaquettes à 55000 /mm^3^), CRP (42mg/l), coproculture (absence de germe entéropathogénes spécifiques), ECBU (stérile), échographie abdominale (sans particularité), radio thorax (sans particularité), des hémocultures ont été demandées. L'enfant a été hospitalisé, réhydraté et a été mis sous une biantibiothérapie empirique à base de C3G (50mg/kg/j) et gentamycine 3mg/kg/j en plus d'une transfusion par des culots phénotypés en attendant une documentation bactériologique.

**Figure 1:**
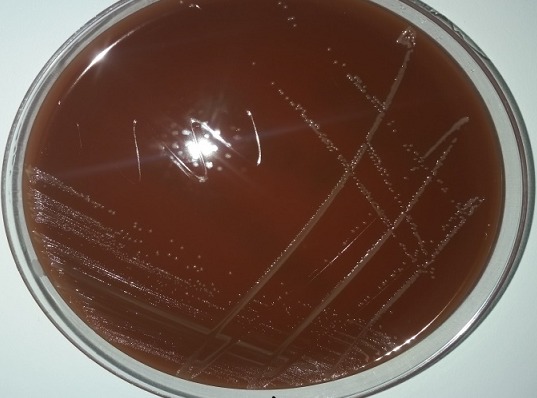
Aspect des colonies de *Kingella denitrificans* après 48h d’incubation

Les hémocultures réalisées les premiers jours d'hospitalisation, avant toute antibiothérapie sont revenues positives après 72heures d'incubation à 37°C sur l'automate d'hémoculture (BACTEC FX BD). L'examen direct a montré la présence de nombreux bacilles à gram négatif polymorphes, de taille variée avec de nombreuses formes courtes, ils sont disposés souvent par paires et sont immobiles à l'état frais. Le repiquage était positif uniquement sur milieux enrichis (Chocolat polyvitex et gélose columbia au sang frais) après une incubation à 37°C en atmosphère aérobie avec 5% de CO2, et la pousse était lente sous forme de très petites colonies translucides (Figure 1). Ces colonies étaient oxydase positive, catalase négative, uréase négative et indol négative, n'hydrolyse pas la gélatine, réduit les nitrates en nitrites, fermente le glucose et le maltose mais pas le xylose (Tableau 1). Une identification automatisée (BD phoenix) et manuelle (Galeries API NH) ont permis de confirmer l'espèce *Kingella denitrificans*. L'étude de la sensibilité aux antibiotiques a été réalisée selon la technique de diffusion des disques en milieu gélosé, et l'interprétation a été faite selon les normes du Comité de l'antibiogramme de la Société française de microbiologie (CA-SFM/EUCAST). La souche était sensible aux antibiotiques suivants: bêtalactamines (amoxicilline, amoxicilline acide clavulanique, céfalotine, céfotaxime, imipenème) - aminosides (amikacine, gentamicine, tobramycine) - Chloramphénicol - Tétracycline - Erythromycine, Pristinamycine - Acide nalidixique - Triméthoprimesulfaméthoxazole. Le test chromogénique a montré que la souche isolée ne produisait pas de bêtalactamase. Au total, les résultats des caractères morphologiques, culturaux et biochimiques ont permis de porter le diagnostic d'une bactériémie à *Kingella denitrificans*. Le traitement a été réajusté ensuite en fonction des résultats de l'antibiogramme (amoxicilline + gentamycine) et une amélioration clinique et biologique a été obtenue avec notamment le retour progressif de l'apyrexie.

**Tableau 1:** Les caractéristiques biochimiques de Kingelladenitrificans

Caractères biochimiques	résultat
Catalase	Negative
Oxydase	Positive
Gélatinase	Negative
Uréase	Negative
Indole	Negative
Réduction des nitrates en nitrites	Positive
Glucose, maltose	Positive
Galactose, lactose, saccharose, xylose, mannitol, sorbitol	Negative

## Discussion

Le genre Kingella a été décrit par Henriksen et Bovre en 1976 [2], il est connu par sa croissance lente et fastidieuse. C'est un cocco-bacille à gram négatif aérobie ou anaérobie facultative (pousse mieux en aérobiose qu'en anaérobiose). Deux types de colonies sont habituellement distingués sur gélose au sang: les unes s'étalent, creusent la surface de la gélose (le diagnostic différentiel avec Eikenella peut se poser); les autres sont lisses et convexes sans autres propriétés. Kingella appartient à la famille des Neisseriaceae, qui comprend également les genres Neisseria, et Ekinella. Les quatre espèces connues du genre kingella sont: *Kingella kingae, Kingellaindologènes*, *Kingella denitrificans* et *Kingellaoralis*. Ces espèces saprophytes des muqueuses et du tractus respiratoire supérieur sont peu fréquemment isolées, mais sans doute méconnues la plupart du temps. Parmi ceux-ci, *K. Kingae* est l'espèce la plus rapportée en pathologie humaine. L'incidence des infections dus à *K. Kingae* a été signalée avec une fréquence croissante au cours des dernières années. Les plus rapportées en matière des infections dues à *K. Kingae* sont les abcès, la méningite, l'ostéomyélite, Les infections ophtalmiques, la cellulite, l'épiglottite et les infections de la cavité péritonéale [3-5]. Bien que *K. denitrificans* ne soit pas une bactérie pathogène spécifique, elle occasionne parfois de sérieuses infections opportunistes. Il s'agit notamment de l'endocardite, de l'empyème, la chorioamnionite et la maladie granulomateuse Secondaire au sida [6-9]. On ne connait pas les facteurs qui contribuent à la virulence de cet organisme. Cependant, une étude antérieure a montré que *K. denitrificans* possède des pillis de type IV et des phénotypes qui sont associés à cette virulence [10]. L'immunodéficience est susceptible d'avoir un rôle dans la pathogenèse des infections dues à cet organisme. Au moins 6 des patients qui ont été décrits dans la littérature ayant une infection a *Kingella denitrificans* avaient une immunodépression : 2 patients avec un lupus érythémateux systémique, 1 patient avec une cirrhose du foie, 1 patient recevant des corticostéroïdes, 1 patient avec leucémie lymphocytaire aiguë, et 1 patient avec le sida [6-9]. La plupart des tests de sensibilité du genre Kingella aux antimicrobiens ont été basés sur les résultats des tests des isolats de *K. kingae*, et ont indiqué une sensibilité aux beta-lactamines, aux aminoglycosides, au chloramphenicol et au triméthoprime-sulfaméthoxazole. Parmi les six cas signalés d'endocardite causés par *K. denitrificans*, cinq ont été traités avec succès avec de la pénicilline, de l'ampicilline ou de ces molécules associées à un aminoglycoside pendant 4 semaines [1,6]. L'utilisation de céphalosporines a entraîné une bonne réponse clinique dans les cas d'infection due à Kingella [5], mais ceux-ci ont été remplacés par la pénicilline ou l'ampicilline pour l'achèvement de la thérapie ou utilisés comme thérapie alternative dans le cadre de l'allergie à la pénicilline.

## Conclusion

La fréquence d'isolement de *Kingella denitrificans* reste sous-estimée dans notre contexte. Ainsi, et dans un contexte d'immunodépression, Les cliniciens et les microbiologistes doivent penser davantage à ce germe qui est récemment reconnu responsable d'infections invasives opportunistes qui surviennent essentiellement sur des patients fragilisés.

## Conflits d’intérêts

Les auteurs ne déclarent aucun conflit d'intérêts.
